# Impact of positive selection technology on seed yam productivity

**DOI:** 10.1016/j.heliyon.2024.e30397

**Published:** 2024-04-27

**Authors:** Jonas Osei-Adu, Robert Aidoo, Simon Cudjoe Fialor, Stella Ama Ennin, Kingsley Osei, Bright Owusu Asante, Gideon Danso-Abbeam

**Affiliations:** a Council for Scientific and Industrial Research - Crops Research Institute (CSIR-CRI), Kumasi, Ghana; b Department of Agricultural Economics, Agribusiness and Extension, Kwame Nkrumah University of Science and Technology, Kumasi, Ghana; c Department of Agribusiness, University for Development Studies, Tamale, Ghana

**Keywords:** Positive selection technology, Yield, Yam seed, Ghana, Nigeria

## Abstract

Positive Selection (PS) technique has been shown to reduce virus infection and increase yields, however there is insufficient empirical evidence on how this technology affects seed yam farm productivity. This study employed Propensity Score Matching (PSM) technique to evaluate the impact of PS on seed yam yields of 368 farmers randomly selected from Ghana and Nigeria.

The findings showed that educational attainment, distance from the farm to the nearest market, cropping patterns, and other factors influenced farmers' adoption of PS. Furthermore, the adoption of PS technology resulted in a 16.98 % boost in farm productivity for PS seed yam farmers compared to their productivity without the technology. It is of the utmost importance that PS adoption be supported by developing tailored training materials for farmers to improve their use of the PS technology.

## 1 Introduction

One of the major challenges to yam production over the years has been inadequate access to quality seed yam 1 as a result of the massive virus infections. According to Ref. [2], viruses are a major constraint to yam production and a threat to food security. This is emphased by Ref. [3] with the view that yam viruses in the tropics are of great concern to farmers as they cause important diseases. According to Ref. [4], recycling of diseased and pest-infested seed yam (*Dioscorea* spp.) has resulted in drastic yield reduction in ware yam production. This has been as a result of the transmission of virus from seed yam into the field of ware yam leading to yield reduction.

Prominent among these viruses is the Yam Mosaic Virus (YMV) which can lead to yield losses of about 80 % [5]. As part of the study of [[6], [7]] on the impact of YMV on tuber yields, they reported a yield loss of 53 % from their controlled experiment and between 26 % and 89 % for the field trials. These statistics clearly show the devasting effect of YMV on seed yam quality and its subsequent effect on yam.

Positive Selection (PS) is the identification and tagging of healthy-looking plants from which seed yam is harvested. It involves the physical examination of plants at the vegetative stage for viral disease symptoms and using tubers from apparently symptomless plants as planting materials in seed yam production [3]. The farmer at the vegetative stage carefully selects healthy looking plants and tag them with ribbons, rope, perks or anything possible. These tagged plants are later harvested and stored as seeds for planting with the hope that they are virus free. This practice became popular in potato production in Kenya after an extensive work by the International Potato Centre (CIP).

Positive Selection (PS) as a virus mitigation strategy has been identified as a solution to reduce the spread of viruses and other diseases in root and tuber production. According to Ref. [8] PS technology plots from their potato trials gave an average yield of 14.2 t/ha which was significantly higher than the 11.8 t/ha for farmer saved seed. Similarly [5] indicated yields from positively selected plants were significantly higher from non-positive selected plants such that positively selected plants gave a yield of 6.03 t/ha compared to 4.99 t/ha for non-PS plants for their yam on-station trial. According to Ref. [4], the incidence of YMV was 38 % and 67 % for PS and Non-PS respectively on their 2016 trial. They further reported yields of 7.7 t/ha and 5.9 t/ha for PS and Non-PS plots respectively. Similarly [3], reported a viral disease incidence of 35.70 % and 54.11 % in Ghana for PS and Non-PS respectively. They further reported disease severity rating of 1.51and 2.80 for PS and Non-PS respectively.

The above statistics clearly show the ability of the PS technology as a viral mitigation measure and its effect on productivity. This was the basis on which the Community Action in Improving Farmer Saved Seed Yam project (CAY-Seed) did introduce the PS technology in Ghana and Nigeria from 2014 to 2018. The objective was to generate empirical evidence on the ability of the PS technology to improve yields. According to Ref. [9], the Total Factor Productivity of using PS technology was 0.758 as compared to 0.700 for using only the other agronomic practices leading to a technology gap of 2.3 %. The net return for using PS technology according to Ref. [10] was US$ 3417.98/ha which is significantly higher than $1795.58/ha for non-PS. These studies clearly indicate the viability of the PS technology. What is currently missing in literature is the information about the impact of PS technology on farm productivity (yield). This paper therefore provides empirical evidence on the impact of PS technology on farm productivity which will serve as a guide to the promotion and adoption of the technology. Yams are essential for guaranteeing food security in both Ghana and Nigeria. Assessing the impact of PS on the seed yam yield of communities who are heavily reliant on yam is crucial for the long-term sustainability of agricultural development in these countries.

## 2 Methodology

### 2.1 Source of data and sampling

The population for this study was 480 seed yam farmers across Ghana and Nigeria within 16 communities where the CAY-Seed project was implemented. The CAY-Seed project was a pilot project from 2014 to 2018 implemented across Ghana and Nigeria with the objective of testing the effectiveness of the PS technology in seed yam production.

Using Randomized Control Trial (RCT), the project selected 16 communities across Ghana and Nigeria and divided these communities into treatment (PS) and control (Non-PS). The 8 communities in Ghana and Nigeria classified as treated communities were introduce to the use of the PS technology with planting on ridges, row planting and the use of neem leaf powder as a package. Control communities on the other hand were introduce to only planting on ridges, row planting and the use of neem leaf powder without PS technology as part of their package. With socio demographics and other characteristics similar, the only difference between the treated and control communities were the use of the PS technology which was the variable of interest for this study.

The minimum required sample for this study using the formulae as proposed by Ref. [[11], [12]] was 218 based on the population of 480 seed yam beneficiaries of the CAY-Seed Project. However [13], proposed oversampling of 40–60 % to account for low response rate and uncooperative subjects. As a result, 368 CAY-Seed project beneficiaries from Ghana and Nigeria were sampled for this study comprising 186 who used the PS technology and 182 who used only the other agronomic practices (planting on ridges, row planting and the use of neem leaf powder). This led to a response rate of 89.67 % which is higher than the 60 % desirable response rate as proposed by Ref. [14].

**Fig. 1:**
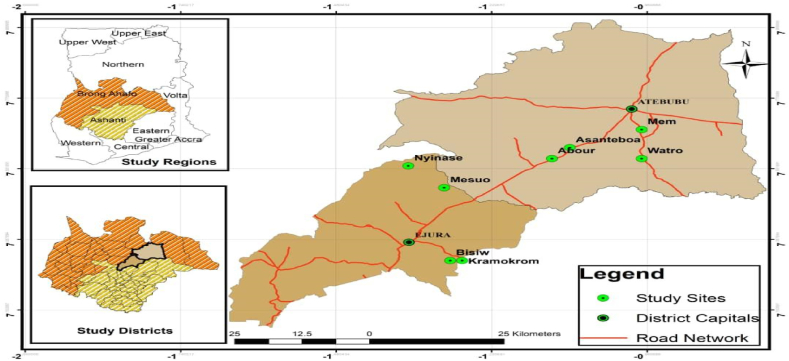
Map of Ghana showing the study area.

**Fig. 2:**
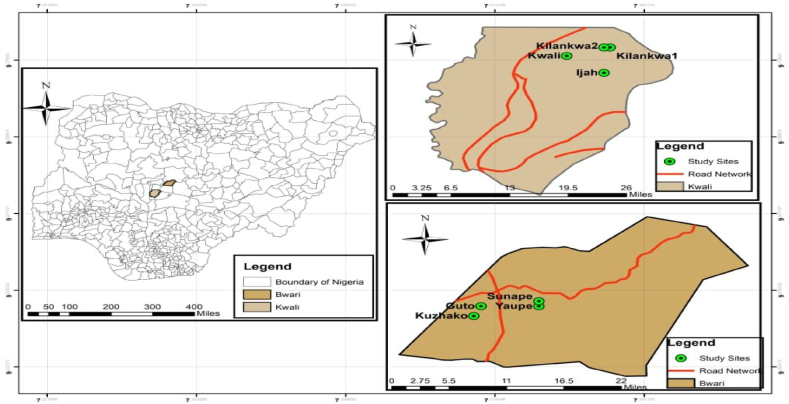
Map of Nigeria showing the study area.

The sample selection was through the use of the multi-stage sampling technique. The 1st stage was the purposive selection of Ghana and Nigeria, where the CAY-Seed project was implemented. The 2nd Stage was a census of two project districts (Ejura-Sekeyedumase and Attebubu) for Ghana (Fig. 1) and Bwari and Kwali Local Area Councils in Nigeria (Fig. 2). The 3rd stage was a census of 16 project communities across Ghana and Nigeria, with the final stage being the simple random sampling of 23 beneficiaries from project communities (PS and Non-PS). The simple random sampling was undertaken using the Excel randomization function based on a project beneficiary list obtained from the CAY-Seed project.

### 2.2 Analytical framework

The use of seed yams from PS materials lead to an increase yield due to the virus-free nature of such materials. Using PS materials however requires extra labour for virus-free plant identification and tagging which comes at a cost. The rational farmer is therefore likely to adopt the PS technology when the net benefit exceeds the extra cost of labour. Based on [15], the net benefit of the ith farmer who uses PS technology can be expressed as PS_i_* and that of the farmer who does not use PS technology as NPS_i_*. The rational farmer is therefore likely to adopt the PS technology when PS_i_*> NPS_i_*. However, the benefit from using the PS technology is unknown but rather the characteristics and attributes of the farmer's choice. That is PS_i_* is unobservable but can be expressed as a function of observable elements in a latent variable related to a set of socioeconomic and technological characteristics. This can be expressed as;

(1) PS_i_* = *αX*_*ij*_*+μ*_*i,*_ PS_i_ = *1[*PS_i_* *>0]*

Where PS_i_ is a binary variable with a value of 1if the farmer uses PS Technology and 0 if otherwise, α is a set of parameters to be estimated, X_ij_ represents a set of demographic, socio-economic and farmer characteristics with μ_i_ as the error term which is assumed to be normally distributed. The probability of a farmer using PS technology can therefore be expressed as:

(2) Pr (PS_i_ = 1) = Pr (PS_i_*>0) = Pr (μ_i_ > -αX_ij)_ = 1-Ω (-αX_ij_)

where *Ω* is the cumulative distribution function for μ_i._ A standard Probit model was therefore employed in the estimation of the parameters in equation (2).

When examining the influence of PS adoption on seed yam yield, it would be erroneous and overly simplistic to assume that differences in yield between PS farmers and non-PS farmers are solely due to PS adoption. When utilizing experimental data and having knowledge of the counterfactual problem, as highlighted by 16, the matter of causal inference becomes irrelevant. Nevertheless, the problem of establishing causal relationships is inherent in cross-sectional data, as the counterfactual scenario remains unknown. The issue of bias in impact evaluation analysis when using observational data has been tackled in the literature through two commonly employed econometric methods: propensity score matching (PSM), which employs propensity score techniques, and endogenous switching regression (ESR), which uses instrumental variable techniques. ESR explicitly incorporates the modelling of endogeneity and self-selection bias by considering the potential for individuals to choose to be part of treatment groups depending on observed or unmeasured attributes. In addition, ESR recognizes the possible presence of latent variables that may impact both the treatment and outcome variables. However, it is important to recognize that ESR does have some limitations, including the potential for model misspecification. A significant drawback of the ESR approach is the challenge of identifying suitable instruments for estimating the model, as well as the requirement to assume a linear functional form. This assumption implies that the estimated parameter for the control variable is identical for both PS farmers and non-PS farmers 17. Nevertheless, as pointed out by 18, the coefficient may vary. Therefore, this assumption may be invalid.

In contrast to the aforementioned parametric methodologies, PSM does not necessitate any assumptions regarding the functional form that specifies the link between the PS variable and yam seed yield, nor the endogeneity of covariates, in order to estimate the causal impacts of the outcome variable. Indeed, all the variables in the model have the potential to be endogenous 17. The goal of propensity score estimation is to attain equilibrium in the observed distribution of variables between the treated group (PS farmers) and the untreated group (non-PS farmers). However, the Conditional Independence Assumption (CIA) imposes a limitation to the PSM approach. According to the CIA assumption, adoption of PS is not influenced by the possible outcome (yield), given a specific set of observed covariates. According to 19, the outcome of the PS farmers and non-PS farmers may differ in a systematic way, even after considering observable factors. These discrepancies may occur when individuals choose to be in the PS group based on traits that are not noticed. Nevertheless, 18 pointed out that assuming selection based on observable characteristics (in the case of PSM) is less stringent than assuming a specific functional form (in the case of ESR), and that it can be challenging to uncover strong instruments when using the instrumental variable approach in cross-sectional data analysis. Due to the cross-sectional nature of the data and the challenge of obtaining appropriate and purely exogenous instruments to justify the use of the instrumental variables approach, the study employed the PSM method to evaluate the impact of PS on the yam seed productivity of farmers in Nigeria and Ghana.

The PSM approach as proposed by 20 was used to determine the significant difference between the net benefit of farmers who used seed yams from PS (treated group) and those who did not (control). The second stage involves matching each PS farmer to a Non-PS farmer conditioned on similar characteristics. This was done after the propensity scores had been computed using equation [3]. The average treatment effect on the treated (ATT) was then derived using this process. The ATT was the net impact of PS on the yield or productivity of the PS farmers. The PSM was use to estimate the net yield effect between the PS and non-PS farmers, known as the average treatment effect (ATE). The Average Treatment Effect (ATE) was therefore specified as:

(3) Δ_i_ = Y_PS_-Y_NPS_

where Y_PS_ represents average yield (kg/ha) of farmers who used PS technique and Y_NPS_ for those who did not. To estimate the impact of the PS technique from (3), the observed yield of the farmer can be expressed as;

(4) Y_i_ = Di (Y_iPS_) + (1-D_i_) Y_iNPS_ D = 0,1

Denoting *Pr* as probability of observing a farmer with D = 1, the ATE can be expressed as:

(5) ATE = Pr [E (Y_iPS_ |D = 1) - E(Y_iNPS_|D = 1)] + (1-Pr) [E (Y_iPS_ |D = 0) - E(Y_iNPS_|D = 0)]

The causal inference problem from (5) resulting from the observed counterfactual *[E (Y*_*iPS*_ |*D* = *0) - E(Y*_*iNPS*_*|D* = *0)]* can be resolved with the PSM estimation technique. PSM summaries pre-treatment characteristics of each subject into a single index variable and proceeds to match similar individuals using propensity scores. PSM which defines the probability of assignment to treatment condition on pre-treatment variables is expressed as;

(6) *p(X) = Pr[D = 1|X] = E[D|X]; p(X) = Ω {h (X*_*i*_*)}*

where Ω {.} can be a normal or logistic cumulative distribution and X as a vector of pre-treatment characteristics*.* Two assumptions were satisfied in the use of the propensity scores. That is the CIA which requires that the common variables that affect treatment assignment and treatment specific outcomes be observed. The second assumption was that the ATT is only defined within the region of common support. The ATT effect was therefore specified as;

(7) *ATT* = E{*Y*_*iPS*_ - *Y*_*iNPS*_*|D = 1}*

(8) *ATT* = E [E{*Y*_*iPS*_ - *Y*_*iNPS*_*|D = 1, p(X }]*

(9) *ATT* = E [E{*Y*_*iPS*_ |*D* = *1, p(X) }-E{Y*_*iNPS*_ |*D* = *0, p(X)}|D* = *1*

### 2.3 Empirical model estimation

The empirical model was estimated using a single stage PSM such that;

(10) $$Y_{i}=\beta_{0}+\beta_{1}Sex+\beta_{2}Age+\beta_{3}Educ+\beta_{4}Hsize+\beta_{5}\;\mathrm{Exp}+\beta_{6}Ext+\beta_{7}Dist+\beta_{8}Crop+\varepsilon_{i}$$

Where Y_i_ denotes seed yam yield (t/ha); Sex of the seed yam farmer measured as a dummy (1 = Male and 0 = if otherwise); Educ being the formal educational status of the seed yam farmer (number of years spent in school); Hsize as the household size; Exp refers to experience in yam cultivation (years); Ext being extension contact (number of contacts per year); Dist being distance from the farm to the nearest market (km); Crop being the cropping system use by the seed yam farmer measured as a dummy (1 = if the farmer uses mono cropping and 0 = if otherwise); β_*0*_ is the constant term, β_*1*_ – β_*8*_ are parameters that were estimated and $\varepsilon_{i}$ as the error term.

## 3 Results and discussion

### 3.1 Descriptive statistics

Summary descriptive statistics of the respondents in terms of their socio economic and farm level characteristics are presented in Table 1. Statistically, age, education, distance and type of the cropping system for farmers who used the PS technology (PS) were different from those who did not use the PS technology. These variables therefore were critical in determining the farmers’ decision to adopt PS technology. From Table 1, males dominated the sample with a mean of 0.65 and 0.67 for PS and Non-PS communities respectively. This is consistent with most farming systems as documented in by Refs. [21], [22], [23], [24]. This male dominance emanates from the fact that yam is a traditional crop such that females in some communities are forbidden from entry into yam farms. Coupled with this is the high capital requirement for seed, labour and other inputs. Females most often have limited access to these resources explaining their limited participation in yam seed production. This is sometimes exacerbated by their inability to provide household labour as a cost mitigation measure.

**Table 1:** Summary of descriptive statistics.

Variable	Variable definition	Positive Selection		Non- Positive Selection		Mean Difference	t-value
		Mean	SD	Mean	SD		
Sex	Dummy (1 = Male, 0 = if otherwise	0.65	0.48	0.67	0.47	0.03	0.54
Age	Years	46.17	12.66	37.12	10.30	−9.05	−7.11***
Education	Years of formal education	3.72	4.71	7.25	5.08	3.52	6.23***
Household size	Number of persons eating from the same pot	8.78	4.85	8.80	5.44	0.12	0.03
Experience	Years in yam cultivation	18.40	13.17	19.25	11.29	0.85	0.63
Extension contact	Number of contacts per year	7.50	5.61	7.53	7.02	0.03	0.04
Distance	Distance from the farm to the nearest market (km)	22.87	11.96	8.35	7.36	−14.52	−13.32***
Cropping system	Dummy (1 = Mono cropping, 0 = If otherwise)	0.41	0.49	0.25	0.44	0.16	−3.15***

Farmers within the study areas were generally in their labour-active age-bracket with average age of 46 years and 37 years for PS and non-PS communities respectively. The difference in age was however significant suggesting that farmers in non-PS were more youthful than PS communities. This result is contrary to Ref. [25] who stated that yam farmers in the Guinea Savanaah zone of Nigeria were aged 41–70 years. This, however, confirms [26] who stated 63.3 % of yam farmers in Edo State of Nigeria were between the ages of 20–50 years. It further confirms [27] who stated large yam farms in Ghana were mainly owned by young men aged 18–35years.

The years spent in school was low with an average of 4 years and 7 years for the PS and non-PS farmers, respectively. The difference between treatments (PS and non-PS) was significant with a mean difference of 4 years. This confirms [28,29] who indicated that 55 % and 42 % respectively of yam farmers in their respective studies had no formal education. This low level of formal education has the tendency of affecting dissemination of improved seed yam technologies and other management practices. Farmers with more years of formal education are more likely to adopt improved technologies. Technology dissemination programmes on seed yam production must therefore develop very simple participatory approaches that can easily be understood by the less formally educated.

Average household size within PS and non-PS communities was approximately 9 persons per household which did not differ between treatments. This confirms the fact that most yam farmers have large family size as documented by Refs. [30,31,32]. This large household size provides farm labour as a complement to hired labour. Seed yam production is a labour-intensive activity and reliance on hired labour mostly becomes a challenge in terms of its availability and cost. The alternative is therefore family labour which is readily available. Seed yam farmers in both PS and non-PS communities were highly experienced with an average of 18 years and 19 years in yam production, respectively. The mean difference of 0.85 was not statistically significant. The high level of experience resonates with findings by Refs. [33,34,26]. This presents a good opportunity for technology dissemination since more experience farmers are more likely to adopt improved technologies. This normally emanates from their several encounter with development practitioners and possible participation in field demonstrations.

Extension contact was measured as the frequency of visits per year which was relatively high for both PS and non-PS technology farmers. The average extension visit per year was 8 for both PS and non-PS farmers. This was contrary to findings of [11] who reported 74.2 % of the respondents had no extension contact in their study. The high frequency of extension contact provides a very good channel for technology dissemination.

The distance from the farm to the nearest market is very important in price determination and access to market. Farmers in PS communities on the average had to travel about 22.87 km before accessing their nearest market. This is very far compared to what [33] reported for adopters and non-adopters (9.2 km and 13.68 km) respectively for improved yams varieties in Ghana. Farmers in the non-PS communities on the other hand had relatively shorter distance of 8.35 km which is in agreement with [33]. This far distance for PS communities however is in line with [35] who reported majority of yam farmers in Kogi State of Nigeria travel about 11–20 km from their farms to the market. These far distances can lead to huge post-harvest losses with implications on food security, profitability and ultimately livelihoods.

The descriptive results of Table 1 indicates that seed yam farmers in the study areas were more tilted towards mixed cropping than sole cropping. With means of 0.41 and 0.25 for PS and non-PS farmers respectively, it is clear that most farmers in these communities were mix crop farmers. With a mean difference of 0.16 which was statistically significant, this result show that mix crop farmers stand to benefit more in using the PS technology.

### 3.2 Determinants of positive selection

The findings of the estimated probit model analysing the factors influencing the adoption of PS by seed yam farmers are displayed in Table 2. The probit model is highly statistically significant at a significance level of 1 %, as evidenced by the likelihood ratio value (*LR Chi-square (9) = 33.62; p = 0.000*). From Table 3, sex of the farmer, educational attainment, household size, distance from the farm to the nearest market and type of cropping systems are the significant socio economic and farm level determinants of PS technology adoption.

**Table 2:** Determinants of positive selection technique.

Variable	Coefficient	S. E.
Constant	0.046	0.369
Sex	0.510***	0.162
Age	−4.963	10.544
Education	−0.033**	0.016
Household size	−0.824*	0.485
Experience	0.217	0.545
Extension contact	−0.863	0.553
Distance	1.679***	0.626
Cropping system	−0.422***	0.159
Location	0.0787	0.184
LR chi2(9)	33.62***	
Log likelihood	−211.19264	
Pseudo R2	0.0737

**Table 3:** Impact of PS technology on farm productivity.

	Treated	Control/counterfactual	Difference	S.E.	t-stats
ATE	5.374	4.459	0.916	0.110	8.30
ATT	5.381	4.599	0.781	0.148	5.28

The probit model result indicates that male farmers are more likely to adopt PS technology than their female colleagues (Table 2). This is may be due to the relatively labour-intensive nature of PS technology in terms of disease-free plants identification, tagging and harvesting. Men dominate the yam industry to the extent that, according to a study by Ref. [36], roughly 85 % of household heads are male. In a related study [23], found that men dominate home decision-making and resource management, which puts them in a stronger position to embrace PS technology use given its labor-intensive nature. Men provide a greater amount of work than women in yam production, which is why they are more likely to embrace the PS technology [37].

The effect of education on the likelihood of adopting PS technology was significant and negative (Table 2). This is may be because highly educated farmers are more inclined to choose advanced techniques such as tissue culture and aeroponics for obtaining clean seeds. The labour-intensive nature of PS technology may not necessarily appeal to them. This finding contradicts the assertion made by Ref. [38] that education has significant positive effect on adoption. Hence, the promotion of PS technology should be directed towards individuals with lower levels of formal education, since they are more inclined to adopt and derive more advantages from it.

Household size was significant but had an inverse relationship with the likelihood of a farmer using PS technology (Table 2). This was contrary to Ref. [39] who reported a positive relationship between household size and the adoption of improved maize varieties in northern Ghana. This inverse relationship can be attributed to the household structure which might be made of children and elderly. The ability of household size to positively influence adoption depends on the number of economically active persons in the household who can contribute to the labour demands of the household.

Distance to the nearest market had a positive significant effect on the probability of seed yam farmers adopting the PS technology (Table 2). This implies that as the distance to the nearest market increases, seed yam farmers are more likely to adopt the PS technology. This is the case because the PS technology is more convenient for small scale farmers who have less access to distant markets. The cost of input acquisition and sale of produce becomes expensive as the distance increases which is a disincentive to small scale farmers who are most likely to adopt the PS technology and hence the positive relationship. This is contrary to Ref. [40] who indicated an inverse relationship between distance to the nearest market and adoption of white yam in Ghana.

The effect of cropping system on the likelihood to adopt the PS technology was significant and negative for mono cropping (Table 2). This could be due to the ability of the technology to reduce the viral load on the field which is most likely to benefit other crops in a mixed cropping system. Farmers involved in mixed cropping system are therefore more inclined to adopt the technology as compared to those in mono cropping. Targeting mix crop farmers for promotion and dissemination would therefore be very important in increasing adoption.

### 3.3 Impact of positive selection on farm productivity

**Fig. 3:**
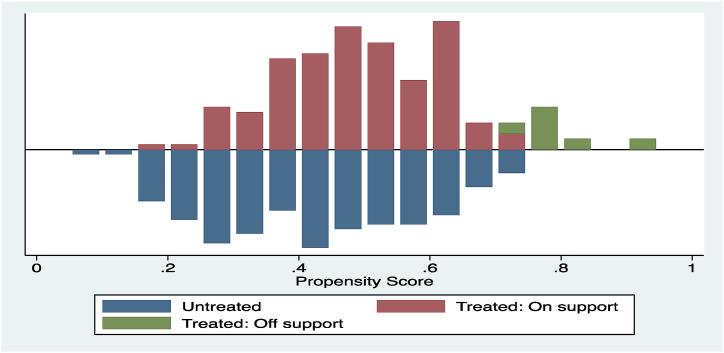
Distribution of propensity scores and presence of common support. Regarding the treated, on-support implies that PS farmers have an adequate control group in the form of non-PS farmers. Treat: Off-support farmers are those who received PS treatment but lacked a relevant control group (non-PS farmers).

After predicting the propensity scores for both PS farmers and non-PS farmers, the study carried out certain diagnostic tests in order to check the quality of the matching procedure. This was done in order to estimate the impact that PS technology had on the treated yam seed farmers with PSM. Fig. 3 presents a density distribution of the propensity scores for yam seed farmers who have received the PS treatment as well as those who have not received it. According to the figure, there is a significant overlap in the distribution of the propensity scores for both PS farmers and non-PS farmers. This is something that is readily apparent. With just 18 (4.89 %) of the treated yam seed farmers being lost, the common support requirement is satisfied, indicating that the condition is satisfied. The histogram is divided into two sections: the upper half shows the propensity score distribution of PS farmers, and the bottom section shows the distribution of non-PS farmers. On the vertical axis, often known as the y-axis, information regarding the distribution densities of the scores is displayed.

It is important to note that the reliability of the common support condition is contingent upon the degree to which the matching techniques are able to build a similarity between the treatment group and the control group based on the covariates.

The results of the estimations of the average impact of the adoption of PS technology on the yield of the treated yam seed households are presented in Table 3, which describes the findings. The study relied solely on observations from the common support zone in order to arrive at an estimate of the ATT. The propensity score of the non-PS farmers in the region of common support is not lower than the minimum propensity score of the PS farmers, and the propensity score of the PS farmers is not higher than the maximum propensity score of the non-PS farmers (Heckman et al., 1997; Becker and Ichino 2002). This is the case in the zone of common support. According to Becker and Ichino (2002), there is a possibility that the accuracy of the estimations could be improved by virtue of the imposition of a common support condition.

The estimations from Table 3 indicates that the average farm productivity of using the PS technology for the entire population was 5.37 t/ha as compared to 4.60 t/ha for using only the other agronomic practices. Though contrary to Ref. [41] who reported an average seed yam yield of 23.8 t/ha when planted early using 30g of seed from their on-station evaluation, this led to a productivity increase of 0.916 t/ha which was significant at 1 % (Table 3) and represented 20.54 %. The impact estimation results further confirmed that adoption of PS technology led to about 0.781 t/ha increase also significant at 1 % (Table 3). This means PS seed yam farmers had 16.98 % more yield than what they could have had without PS technology. This yield increase was as a result of the virus free nature of the positively selected seed yams.

## 4 Conclusions and recommendations

In this study, yam seed producers' decisions about the use of PS technology and the impact those decisions had on yam seed productivity were investigated. The likelihood of farmers adopting PS technology is higher when the farmer is male and when the distance from their farm to the nearest market center is greater. However, farmers were dissuaded from adopting PS technology due to factors such as higher educational levels, larger household sizes, and practices that involved mixed cropping. According to the PSM estimations, the adoption of PS technology results in significant increases in yields. In particular, the adoption of PS technology resulted in an increase in yield that was approximately 20.54 % higher than the average for the total population that was sampled. Adopters of PS technology had around 16.98 % more than they would have had if they had not adopted the technology. The fact that seed yams produced using PS technology are devoid of viruses is a crucial factor that contributes to this accomplishment. As a result, this technology has the potential to play a very significant part in reducing the impact of viral infections on the development of seed yam varieties.

What is required is to effectively promote its use among smallholder farmers who are mostly vulnerable to the YMV. The creation of the right policy environment to ensure the determinants of the impact of the technology are favourable would be critical. It is therefore recommended that PS technology be adopted as one of the main strategies in any integrated pest and disease management strategy for yam production. Enhanced capacity in effective identification of disease-free materials and tagging would be highly necessary in improving dissemination and adoption.

## Availability of data and material

Data for this article is available from the corresponding author upon request.

## Ethics approval and consent to participate

Informed consent was sought from each participant prior to the conduct of interviews. They were made to understand the rational of the study and assurance of keeping their data private upon which they freely and willingly agreed to be part.

## Consent for publication

As authors we do hereby indicate our consent for publication having played an active role in preparing this manuscript.

## Funding

Data for this study was part of a PhD studies sponsored by the 10.13039/100000865Bill and Melinda Gates Foundation (BMFG) through the Community Action in improving farmer saved seed Yam (CAY-Seed) project by the CSIR-Crops Research Institute (CRI).

## CRediT authorship contribution statement

**Jonas Osei-Adu:** Writing – review & editing, Writing – original draft, Methodology, Formal analysis, Conceptualization. **Robert Aidoo:** Validation, Supervision, Methodology. **Simon Cudjoe Fialor:** Validation, Supervision, Methodology. **Stella Ama Ennin:** Validation, Supervision, Resources, Methodology, Funding acquisition. **Kingsley Osei:** Validation, Supervision, Resources, Funding acquisition. **Bright Owusu Asante:** Validation, Software, Methodology, Formal analysis, Data curation. **Gideon Danso-Abbeam:** Visualization, Software, Methodology, Formal analysis, Data curation.

## Declaration of competing interest

The authors declare that they have no known competing financial interests or personal relationships that could have appeared to influence the work reported in this paper.
